# Palliative chemotherapy: oxymoron or misunderstanding?

**DOI:** 10.1186/s12904-016-0109-4

**Published:** 2016-03-21

**Authors:** EJ Roeland, TW LeBlanc

**Affiliations:** Moores Cancer Center, University of California San Diego, La Jolla, CA 92093 USA; Department of Medicine, Division of Hematologic Malignancies and Cellular Therapy, Duke University School of Medicine, Durham, NC 27710 USA

## Abstract

Oncologists routinely prescribe chemotherapy for patients with advanced cancer. This practice is sometimes misunderstood by palliative care clinicians, yet data clearly show that chemotherapy can be a powerful palliative intervention when applied appropriately. Clarity regarding the term “palliative chemotherapy” is needed: it is chemotherapy given in the non-curative setting to optimize symptom control, improve quality of life, and sometimes to improve survival. Unfortunately, oncologists lack adequate tools to predict which patients will benefit. In a study recently published in BMC Palliative Care, Creutzfeldt et al. presented an innovative approach to advancing the science in this area: using patient reported outcomes to predict responses to palliative chemotherapy. With further research, investigators may be able to develop predictive models for use at the bedside to inform clinical decision-making about the risks and benefits of treatment. In the meantime, oncologists and palliative care clinicians must work together to reduce the use of “end-of-life chemotherapy”—chemotherapy given close to death, which does not improve longevity or symptom control—while optimizing the use of chemotherapy that has true palliative benefits for patients.

Oncologists routinely prescribe chemotherapy for patients with advanced, incurable cancer. This practice is sometimes misunderstood by palliative care clinicians, who tend not to see the many patients who routinely benefit from this “palliative intent” chemotherapy, instead being called to evaluate those who are admitted to the hospital with complications or who are generally faring poorly. Yet there are clear data demonstrating that chemotherapy itself can be a powerful palliative intervention when applied appropriately. Clarification regarding the term *palliative* chemotherapy is critical: palliative chemotherapy is defined as chemotherapy that is given in the non-curative setting to optimize symptom control, improve quality of life (QoL) and, ideally, to improve survival.

Evidence demonstrates that palliative chemotherapy has an established role and effectively achieves these aims for select patients with a variety of metastatic solid tumors. For example, chemotherapy can improve pain, physical function, and longevity in patients with advanced pancreatic cancer, while slowing the rate of decline in appetite and the onset of bothersome symptoms like dyspnea and constipation [1, 2]. Similarly, “targeted” biologic therapy can significantly improve pain, dyspnea, and cough in patients with advanced non-small cell lung cancer [3] and QoL and fatigue in breast cancer [4]. Many other such examples exist in the oncology literature, clearly supporting the role chemotherapy has in improving or maintaining a patient’s QoL and addressing troublesome symptoms.

Despite our best efforts, however, oncologists sometimes get these decisions wrong, and thereby either over-treat a patient who is near death (and thus unlikely to benefit from chemotherapy), or under-treat a patient who might derive real palliative benefits from chemotherapy. While professional organizations such as the American Society of Clinical Oncology (ASCO) have recommendations to aid oncologists’ decision-making about the appropriateness of chemotherapy in patients with advanced cancer [5], the data to guide these decisions remain woefully inadequate. Research in this area is sorely needed, and is nicely embodied in the work of Creutzfeldt et al. recently published in BMC Palliative Care [6].

This line of research is particularly relevant given renewed interest in the appropriate role of chemotherapy in advanced cancer settings. In an analysis of data from the “Coping with Cancer Study,” Prigerson et al. recently published findings that challenge a central tenet of palliative chemotherapy prescribing: the notion that patients with good performance status are those most likely to benefit [7]. To the contrary, in this study chemotherapy was actually associated with worse QoL in more functional patients in the last week of life, and was also not associated with improved longevity. These findings have led some to conclude that patients with advanced cancer should not be offered palliative chemotherapy at all, or that the concept of “palliative chemotherapy” is nonsensical. To be clear, these conclusions reflect a misapplication of the study’s findings, and an over-interpretation of the data.

As dual-trained palliative care physicians and medical oncologists, we are particularly sensitive to both sides of this debate, and have been dismayed to hear some of our palliative care colleagues make broad anti-chemotherapy statements following the publication of the Prigerson et al. article. We know that such statements ignore decades of evidence demonstrating clear palliative benefits of chemotherapy in appropriately-selected patients with advanced cancer, and also ignores some of the important limitations of this study, which did not assess symptoms or QoL during the receipt of chemotherapy (precisely the measures shown to improve in most studies of palliative chemotherapy). We also must note that blanket criticisms of palliative chemotherapy are divisive, and even insulting to oncologists’ good intentions when prescribing palliative chemotherapy.

Rather, we should encourage more collaboration between palliative care and oncology, in light of the many recent demonstrations of improved outcomes resulting from earlier, concurrent palliative care as part of cancer care (even alongside chemotherapy) [8–12]. Rather than drive a wedge between our disciplines, these recent findings should motivate and prompt us to elevate the state of the science to address the difficult clinical challenge at hand: how to identify which patients are most likely to benefit from palliative chemotherapy versus those who may actually be harmed by it.

As recently published in BMC Palliative Care, Creutuzfeldt and colleagues present an innovative and important approach to advancing the science in this area: using patient reported outcomes (PROs) to predict responses to palliative chemotherapy [6]. Here, the investigators evaluated the predictive value of pretreatment QoL and symptom burden for patients receiving palliative chemotherapy. They did so in part by utilizing published estimates of response rates and survival, thereby allowing them to explore whether pre-treatment variables might predict the likelihood of benefitting from treatment. Ultimately, the investigators found that while palliative chemotherapy was often helpful, certain baseline features were associated with its impact on treatment outcomes. For example, patients with worse QoL and symptom burden at baseline were less likely to benefit, while those with better physical functioning were more likely to have objective tumor responses by imaging criteria. Perhaps more interesting, however, is the finding that overall global health status (as measured by the EORTC scale) improved with treatment, even among those cases where survival was not improved. Interestingly, even patients who had no objective tumor response by imaging experienced improved QoL and symptom burden with palliative chemotherapy. These findings resonate with oncologists’ motivations for prescribing chemotherapy with palliative intent: where its goal is sometimes just to help patients live better, even if life is not prolonged.

While these findings must be tested in larger, more robust study designs, they present preliminary data for clinicians currently facing difficult decisions about whether to recommend a course of palliative chemotherapy. With further research in this area, investigators may be able to develop predictive models that can be used at the bedside to inform clinical decision-making about the risks and benefits of chemotherapy for an individual patient with advanced, incurable disease. This is a great first step in an important line of research that has great potential to improve clinical care.

While we wait for evidence to develop further in the area, however, we must advise more clarity in our terminology. Palliative chemotherapy is treatment that is given in the non-curative setting to optimize symptom control, improve or maintain QoL and, ideally, to also improve survival. We should separate this concept from one that is far less controversial: “end-of-life chemotherapy.” Here, we refer to chemotherapy that is given close to death, which does not improve longevity and likely also has no benefits in relieving symptoms or improving QoL; indeed it may even worsen survival or symptoms.

Whether oncologist or palliative care clinician, our shared goal should be to reduce the use of “end-of-life chemotherapy” while optimizing the use of chemotherapy that has true palliative benefits for patients. In other words, we must be careful not to throw the proverbial baby out with the bathwater. Indeed, palliative care specialists must be particularly careful not to assume the role of “anti-chemo police” as we continue building models of concurrent care and effective collaboration. Chemotherapy continues to have an appropriate role in palliating symptoms among selected patients with advanced cancer, and we thank Creutzfeldt et al. for providing another example of how palliative care and oncology can learn from each other to address our mutual goal – improving the lives of patients facing a serious illness.

## Abbreviations

ASCO: American Society of Clinical Oncology
EORTC: European Organisation for Research and Treatment of Cancer
PRO: patient reported outcome
QoL: quality of life
